# Carnosol Improved Lifespan and Healthspan by Promoting Antioxidant Capacity in *Caenorhabditis elegans*

**DOI:** 10.1155/2019/5958043

**Published:** 2019-06-24

**Authors:** Chunxiu Lin, Xiaoying Zhang, Zuanxian Su, Jie Xiao, Muwen Lv, Yong Cao, Yunjiao Chen

**Affiliations:** ^1^Guangdong Provincial Key Laboratory of Nutraceuticals and Functional Foods, College of Food Science, South China Agricultural University, Guangzhou, Guangdong, China; ^2^College of Horticulture, South China Agricultural University, Guangzhou 510640, China

## Abstract

Carnosol, a phenolic diterpene, is one of the main constituents of *Rosmarinus*. It is known to possess a range of bioactivities, including antioxidant, anticancer, antimicrobial, and anti-inflammatory properties. Nevertheless, the antiaging effects of carnosol have received little attention. This study first indicated that carnosol increased the healthspan of *Caenorhabditis elegans* (*C. elegans*). First, compared with the control condition, carnosol treatment effectively decreased ROS accumulation under normal or oxidative stress condition, significantly increased several key antioxidant enzyme activities, and significantly decreased MDA content. Second, carnosol effectively prolonged lifespan under normal and stress conditions and slowed aging-related declines, including mobility, age pigmentation, and neurodegenerative disease, but had no effect on fertility and fat deposition. Finally, carnosol-mediated longevity required the upregulated expression of *sod-3*, *sod-5*, *hsf-1*, *hsp-16.1*, and *hsp-16.2* and was dependent on the *hsf-1* gene. Increased DAF-16 translocation was observed, but *daf-16* was independent of the effects on lifespan induced by carnosol. These results suggested that carnosol might serve as a good source of natural antioxidants, and in particular, carnosol could be explored as a potential dietary supplement to slow aging.

## 1. Introduction

As people continue to age worldwide, prolonged lifespan and improved healthspan have become hot topics. The free radical theory proposes that the uncontrolled formation of oxygen free radicals and an imbalance in antioxidant protection induce numerous age-related diseases [1]. Various studies indicate that the antioxidative effects of natural antioxidants, such as (−)-epigallocatechin-3-gallate, a major polyphenol antioxidant in green tea, can scavenge these reactive oxygen species (ROS) and show efficacy in delaying the process of aging [2].

Carnosol is a natural polyphenol (dietary diterpene) found in plants belonging to the Lamiaceae family (mint family), such as sage, rosemary, lavenders, and oregano [3]. Carnosol is known as a promising anti-inflammatory, anticarcinogenic, antibacterial, and antioxidative agent in both *in vitro* and *in vivo* experimental models [4, 5]. In terms of antioxidative activities, there is a large body of research on the activity of carnosol. Carnosol was more effective at scavenging hydroxyl radicals and protecting DNA than vitamin C and vitamin E [6]. Carnosol had an inhibitory activity against lipid peroxidation and had a promoting effect on antioxidant enzymes in the liver of mice [7, 8]. The biological activity of carnosol is very interesting, but its antiaging effects and underlying mechanisms are still open questions that remain to be elucidated and are worthy of further discussion.

*Caenorhabditis elegans* (*C. elegans*) was the first metazoan organism with a completely sequenced genome, 60-80% of which consists of genes homologous to human genes that are highly evolutionarily conserved [9]. It is estimated that more than 83% of the proteins in the *C. elegans* proteome have human homologs [10]. The cellular complexity and the conservation of disease pathways between *C. elegans* and higher organisms, together with the simplicity and cost-effectiveness of cultivation, make *C. elegans* an effective *in vivo* model [9, 11]. A number of food products and ingredients have been reported to modulate the lifespan and healthspan of *C. elegans*, which has provided a wealth of information for understanding the role of genetics in the modulation of aging. Thus, there are increasing efforts and great concerns that employ *C. elegans* to study the potential bioactivity and molecular mechanisms of natural compounds.

In this research, we used *C. elegans* as a model system to evaluate the antioxidation and antiaging effects of carnosol for the first time. Furthermore, the antiaging mechanism of carnosol was elucidated. Our study might promote the development of carnosol as a potential dietary supplement to retard age-related diseases and promote healthy aging.

## 2. Materials and Methods

### 2.1. Materials and Strains

Carnosol was supplied by Wuling Yangguang Biotechnology Co. Ltd. (Hunan, China, purity ≥ 90%) and stored in ethanol solution at 4°C (90 mM in 100% ethanol). The strains used in this study were Bristol N2 (wild-type); GR1307, *daf-16(mgDf50) I*; CF1553, *muIs84 [(pAD76) sod-3p::GFP+rol-6(su1006)]*; AM140, *rmIs132 [unc-54p::Q35::YFP]*; CL4176, *dvIs27 [myo-3p::A-Beta (1-42)::let-851 3*′*UTR+rol-6(su1006)] X*; PS3551, *hsf-1*(sy441) I; and TJ356, *zIs356 IV (pdaf-16-daf-16::gfp; rol-6)*. PS3551 *[hsf-1(sy441) I]* and TJ356 *[zIs356 IV (pdaf-16-daf-16::gfp; rol-6)]* were provided by Prof. Qinghua Zhou (Biomedical Translational Research Institute, Jinan University, Guangdong Province, China), and the other *C. elegans* strains and *Escherichia coli* strain OP50 (*E. coli* OP50) were obtained from the *Caenorhabditis Genetics Center* (University of Minnesota, Minneapolis, Minnesota, USA). The carnosol stock solution was diluted in the *E. coli* OP50 suspension and spread onto the surface of nematode growth medium (NGM) plates. The control group was treated with vehicle alone (0.2% ethanol), and the control groups for stress resistance, the lifespan of mutants (*daf-16* and *hsf-1*), and paralysis assays were referred to previously published data [12].

### 2.2. Accumulation of Reactive Oxygen Species (ROS)

Unless stated otherwise, eggs isolated with hypochlorite were transferred to the treatment NGM plates and maintained at 20°C in a temperature-controlled incubator according to our previous protocol [13]. Synchronization was performed at the L4 larvae stage (with a characteristic half-moon-like spot (black arrowheads) present in the vulva region [14]). ROS levels were determined using 2,7-dichlorodihydrofluorescein diacetate (H_2_DCF-DA) as described elsewhere [15]. In the normal group, the wild-type worms were cultured on NGM for 96 hours with or without carnosol treatment. The worms in the oxidative stress group were transferred to the NGM containing 10 mM paraquat for 24 hours after normal culture. Briefly, wild-type worms were placed on NGM plates to remove bacteria three times (50 individuals). Subsequently, animals were transferred to a black 96-well plate, and H_2_DCF-DA was added into each well to obtain a final concentration of 50 *μ*M H_2_DCF-DA. The fluorescence intensity was determined utilizing an EnSpire multimode plate reader (PerkinElmer, Waltham, MA, U.S.A.) every 15 min for 6 hours at 25°C (excitation wavelength of 485 nm; emission wavelength of 535 nm). Four independent experiments were performed per treatment.

### 2.3. Antioxidant Enzyme Activities and MDA Content Assay

After treatment for 96 h, more than 600 worms were homogenized. The activities of the antioxidant enzymes superoxide dismutase (SOD, U/mg^−1^ protein), catalase (CAT, U/mg^−1^ protein), glutathione peroxidase (GSH-Px, U/mg^−1^ protein), and malondialdehyde (MDA, U/mg^−1^ protein) were measured with commercially available kits (Nanjing Jiancheng Bioengineering Institute, China) according to the manufacturer's instructions. The experiments were independently performed three times.

### 2.4. Lifespan Assay

For each strain, lifespan analysis was generally performed according to the protocol described in our previous study [13]. The animals that failed to respond to mechanical touch were scored as dead and removed from the plate every two days. The worms that crawled off the plate and died away from the agar were excluded from the analysis. Three independent experiments were performed with a total of 180 individuals.

### 2.5. Stress Resistance Assay

For the oxidative stress resistance assay, wild-type worms were cultured for 6 days before being moved to a plate containing paraquat (final concentration: 10 mM). Survival was recorded every 12 hours until all worms were dead. Synchronized wild-type worms were transferred from 20°C to 35°C on day 4 to evaluate thermotolerance. Survival was recorded every hour until all worms were dead. These assays were repeated three times with sixty worms per assay.

### 2.6. Mobility Assay

The mobility assay was carried out on days 6, 10, and 14. Sixty nematodes were observed, and the locomotivity of class A to class C was quantitatively measured according to previous protocols [16]. Class A animals spontaneously moved. Class B animals did not move until prodded by a metal wire, while class C animals simply moved their head or tail in response to stimulus. The number of head swings from one side to the other in 30 seconds and the number of leftward or rightward body bends in 60 seconds were counted. All experiments were performed in triplicate.

### 2.7. Fertility Assay

Ten wild-type L4 larvae were placed on an NGM plate with or without carnosol treatment to produce progeny. Worms were transferred to a fresh plate every 24 hours until egg production ceased. All plates with eggs were continued to be cultured in the 20°C incubator to verify the eggs hatched. Three independent experiments were performed.

### 2.8. Age Pigment Accumulation

The fluorescence intensities of age pigments were quantified by two different methods on days 6, 10, and 14. First, the level of the age pigment was measured using fluorescence microscopy. The fluorescence microscope was set at 100 times magnification using a GFP filter (exposure time: 390.0 ms; gain: 3x) (Axio Imager Z2, Carl Zeiss Microscopy, Jena, Germany). The fluorescent intensity was quantified using ImageJ software. The second measurement methodology involved the use of a microplate reader [17]. The normalized age pigment score was calculated as the ratio of age pigment fluorescence/tryptophan fluorescence. Each analysis was repeated at least three times.

### 2.9. Body Fat Accumulation

The accumulation of body fat was conducted as previously reported, and the freeze-thaw steps were omitted [18]. The Oil Red O stock solution was prepared in isopropanol (6 mg/mL). The Oil Red O staining solution was freshly diluted in deionized water and filtered. Six-day-old worms were fixed in 4% paraformaldehyde, dehydrated in 60% isopropanol, and then stained. Worms were visualized by a microscope (CX41, Olympus Co., Tokyo, Japan) with a 40x objective lens. The relative staining intensity was analyzed using ImageJ software. This experiment was repeated three times with a total of 45 individuals.

### 2.10. Paralysis Assay

Briefly, egg-synchronized CL4176 worms were treated with carnosol for 34 hours at 15°C. After being transferred to 25°C, the number of paralyzed individuals was counted at 2 h intervals until all the worms were paralyzed. AM140 mutants were recognized as paralyzed when they failed to move forward after being touched in daily evaluations. These assays were repeated three times with a total of 180 individuals.

### 2.11. Gene Expression Analysis by Quantitative Reverse Transcription-Polymerase Chain Reaction (qRT-PCR)

Adult worms at day 3 were harvested, and total RNAs were extracted with a TRNzol Total RNA Extraction kit (TIANGEN). Complementary DNA was synthesized using HiScript® ΙΙ Q RT SuperMix (+gDNA wiper) (R223-01, Vazyme). qRT-PCR was carried out using iTaq™ Universal SYBR® Green Supermix and the CFX96™ Real-Time PCR detection system according to the manufacturer's protocol (Bio-Rad). *act-1* was chosen as the reference gene, and relative quantification of gene expression was performed using the 2^−*ΔΔ*Ct^ method [19]. All primers are shown in Table S1. Samples were run in triplicate.

### 2.12. Quantification of GFP-Labeled SOD-3 Expression

The GFP fluorescence of CF1553 animals was assayed using a microplate reader (EnSpire Multimode Plate Reader, PerkinElmer, Waltham, MA, USA). Fifty 6-day-old worms were transferred to a black 96-well plate containing 50 *μ*L of 10 mM NaN_3_ solution per well, and 200 *μ*L of PBS was also added to each well. Total GFP fluorescence was measured using 485 nm excitation and 530 nm emission filters in four experiments.

### 2.13. The Subcellular Localization of DAF-16::GFP

Approximately 45 randomly selected TJ356 worms from each experiment were anesthetized after 6 days of culture. All fluorescence determinations were performed with a Leica TCS SP8 confocal laser microscope (Leica Microsystems, Buffalo Grove, IL, USA) using a 10x objective lens. Images were acquired with a 488 nm excitation filter and a 500/525 nm emission filter. TJ356 worms were classified as cytoplasmic, intermediate cytoplasmic/nuclear, and strong nuclear translocation. Three independent experiments were performed.

### 2.14. Statistical Analysis

Data are presented as the mean ± SD. Log-rank (Mantel-Cox) tests were executed to analyze the survival curve using GraphPad Prism version 5.00 for Windows (GraphPad Software Inc., San Diego, California, USA). The post hoc comparison (LSD and Duncan tests) with one-way analysis of variance (ANOVA) was performed using SPSS software. Different letters within a column indicate significant differences (*p* < 0.05).

## 3. Results and Discussion

### 3.1. Carnosol Lowered ROS Accumulation in *C. elegans* under Both Normal and Oxidative Conditions

**Figure 1:**
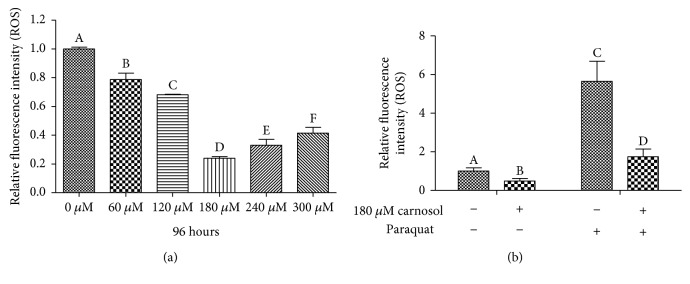
Effects of carnosol treatment on ROS accumulation. (a) ROS levels were significantly reduced after 96 hours of treatment with different concentrations of carnosol, showing a U-shaped dose-response curve. (b) The ROS accumulation in the worms pretreated for 96 hours after exposure to or without exposure to 10 mM paraquat for 24 hours. Carnosol decreased the ROS accumulation of *C. elegans* under normal and paraquat-induced oxidative stress conditions.

ROS is a byproduct of cellular metabolism [20]. *In vivo*, excessive ROS may cause random damage to proteins, lipids, and DNA and may ultimately lead to cancer, aging, and many chronic diseases [21]. To evaluate the influence of carnosol on the level of ROS accumulation *in vivo*, we pretreated synchronized 4-day-old adult N2 worms with different concentrations of carnosol (60, 120, 180, 240, and 300 *μ*M) followed by exposure to the superoxide probe H_2_DCF-DA. As shown in Figure 1(a), the ROS levels of *C. elegans* treated with different concentrations of carnosol showed a significant decrease compared with those of control *C. elegans*. In particular, a decrease of up to 76% was observed in the 180 *μ*M carnosol treatment group compared to the control group. The change in the ROS levels of worms exposed to the concentrations of 60, 120, 240, and 300 *μ*M carnosol was smaller than that of worms exposed to 180 *μ*M carnosol. Therefore, the following experiments were performed with the 180 *μ*M carnosol treatment.

To test whether the beneficial reduction in ROS levels was also present under oxidative stress conditions, we assessed ROS accumulation after the addition of 10 mM paraquat. Paraquat is known to act as an oxidative stressor and capable of inducing the formation of mitochondrial ROS [22]. The results showed that the accumulation of ROS was significantly increased after 24 hours of exposure to paraquat (Figure 1(b)). Interestingly, the worms treated with carnosol showed a reduction in ROS accumulation regardless of the presence or absence of paraquat. Therefore, we concluded that carnosol exhibited a strong attenuation of ROS *in vivo* under both normal and oxidative stress conditions.

### 3.2. Carnosol Treatment Promoted Antioxidant Activity and Decreased MDA in *C. elegans*

**Figure 2:**
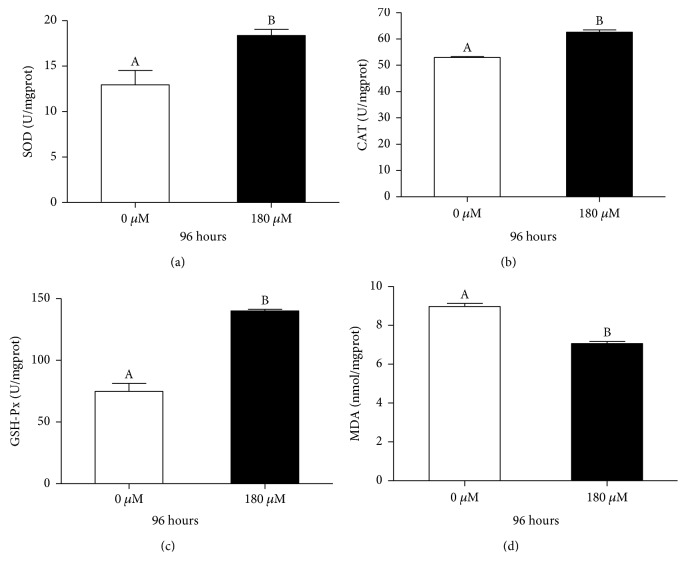
Effects of carnosol treatment on antioxidant enzyme activities and MDA content. (a) SOD activity, (b) CAT activity, (c) GSH-Px activity, and (d) MDA content were detected. These results are expressed as enzyme units.

It is known that oxidative stress caused by excessive ROS can be ameliorated by cellular antioxidant defense systems, which play a critical key role in maintaining a healthy metabolic environment. Antioxidant enzymes are crucial components of the antioxidant defense system *in vivo* [23]. To further explore the effect of carnosol on the antioxidant system, the activities of intercellular SOD, CAT, and GSH-Px were measured. As shown in Figures 2(a)–2(c), the activities of SOD, CAT, and GSH-Px were significantly enhanced by 42%, 18%, and 87%, respectively, after 4 days of treatment with carnosol. In addition, the level of lipid peroxidation, as evidenced by MDA, was significantly decreased by 21% in the carnosol treatment group compared with the control group (*p* < 0.05, ANOVA, Figure 2(d)). Taken together, supplementation with carnosol could enhance antioxidant activity by promoting antioxidant activity and decreasing MDA and ROS accumulation in *C. elegans*.

### 3.3. Carnosol Extended the Lifespan of *C. elegans* under Normal and Stress Conditions

**Figure 3:**
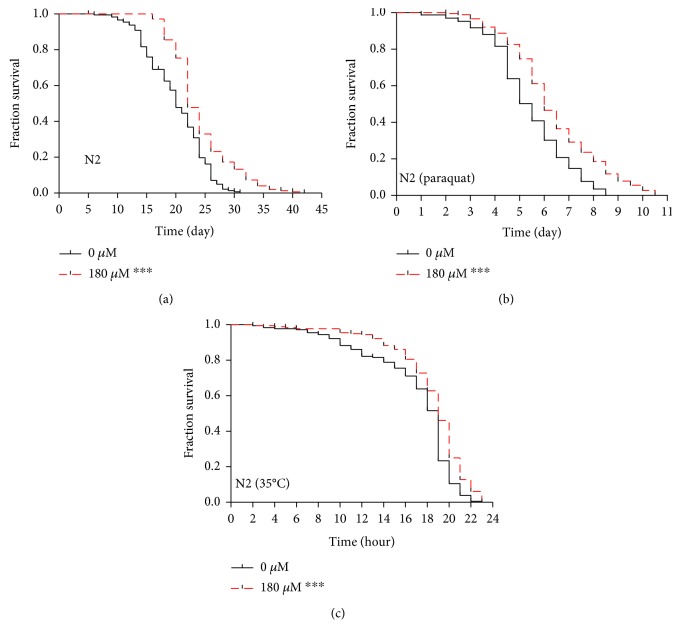
Effects of carnosol on the lifespan of *C. elegans*. (a) Survival curves of N2 worms treated with the control or carnosol are shown. The lifespan of 180 *μ*M carnosol-treated nematodes was significantly prolonged compared with that of control nematodes. (b) Survival curves of carnosol-treated or untreated worms exposed to acute oxidative stress in the days after induction by paraquat (from the sixth day of the life cycle). (c) Survival curves of carnosol-treated or untreated worms in the hours after exposure to high temperatures (from the time of exposure to high temperature stress).

Extensive evidence from experimental studies with cell and animal models suggests that many plant polyphenols with antioxidant activity also have antiaging effects, such as resveratrol, EGCG, quercetin, and anthocyanins [24]. Therefore, we further examined the effect of carnosol on the lifespan of *C. elegans*. As shown in Figure 3(a), we found that compared with the control worms, the worms treated with carnosol not only showed a significantly different survival curve (*p* < 0.0001 as determined by the log-rank test) but also exhibited a significant 19% increase in mean lifespan, a 12% increase in median lifespan, and a 26% increase in maximum lifespan (Table 1).

Compounds that extend normal lifespan might be associated with improved survival under environmental stress [25]. Under oxidative stress conditions, a significant 21% increase in lifespan was observed in carnosol-treated worms compared with control worms (Figure 3(b) and Table 1). Furthermore, the addition of carnosol under heat stress increased the survival of worms by 9% (Figure 3(c) and Table 1). Therefore, carnosol was an effective longevity regulator under normal and stress conditions in *C. elegans*.

### 3.4. Carnosol Promoted the Enhancement of Healthspan in *C. elegans*

A central aim of aging research is to identify strategies that maintain healthspan, the period in adulthood without age-related disease and physical impairment that precedes senescent decline, as long as possible [16]. To investigate the effects of carnosol on healthspan, we determined the effects of carnosol on mobility, reproductive capacity, age pigments, fat accumulation, and neurodegenerative diseases in *C. elegans*.

**Figure 4:**
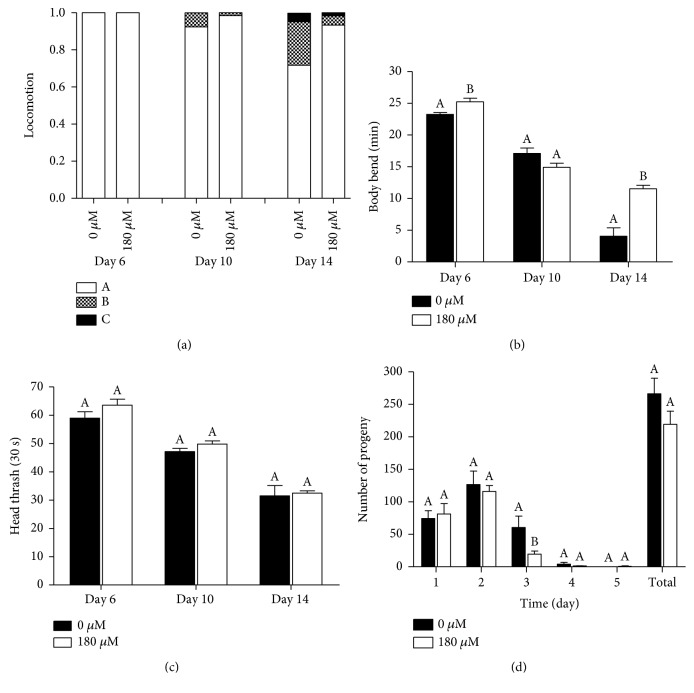
Effects of carnosol on the mobility and fertility of *C. elegans*. Mobility was tested in three ways: (a) the three levels of locomotion; (b) the body bend frequency; (c) the frequency of head thrashing. (d) Time-course distribution of fertility and the total number of progeny produced by the control and carnosol-treated worms are shown.

As an animal ages, mobility progressively decreases, indicating the physical deterioration of muscle [26]. Locomotion was analyzed at the early, middle, and midlate life stages (on days 6, 10, and 14, respectively). As shown in Figure 4(a), the proportion of spontaneously exercising worms in the carnosol treatment group was higher than that in the control group, and the adverse effect of aging on exercise was lower in the carnosol treatment group than in the control group. Additionally, we quantitatively measured body movement by calculating the number of body bends and the head swing frequency to indicate the movement states of parts of the body and the whole body. Compared with the control condition, the carnosol treatment significantly inhibited the decline in body bending that occurred with age, especially in the early and midlate periods (Figures 4(b) and 4(c)). Additionally, carnosol treatment did not significantly change the head swing frequency at the three stages, indicating that carnosol had no side effects on the ability of the head to move (*p* > 0.05, ANOVA). These results indicated that carnosol could effectively improve the mobility of nematodes in the process of senescence.

To investigate the effect of carnosol on the reproductive capacity, the number of progeny produced on each day of adulthood was measured. We found that the number of progeny was reduced in the carnosol-treated group compared with the control group on the third day of spawning (Figure 4(d)). Nevertheless, the results did not show any significant differences in the total number of offspring between the control and treated worms or any inhibition during the reproductive peak of the previous two days. Therefore, the longevity mediated by carnosol was not accompanied by a reduction in fertility.

**Figure 5:**
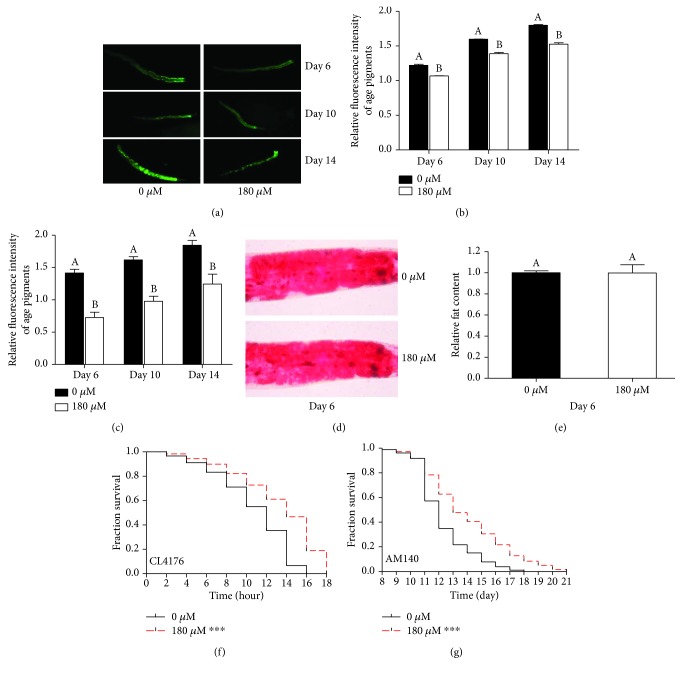
Effects of carnosol on other physiological functions of *C. elegans*. (a) Fluorescence representation of age pigments is shown. (b) Carnosol effectively suppressed age pigment accumulation on days 6, 10, and 14. (c) Similar results were obtained by measuring the relative fluorescence of age pigments using a microplate reader. (d) Representative images of lipids stained with Oil Red O are shown. (e) Quantitative analysis of Oil Red O staining intensity revealed no significant change in fat content. (f) Curves of the A*β*-induced paralysis of CL4176. The number of paralyzed CL4176 animals was counted every 2 hours. (g) PolyQ-dependent paralysis curve of the AM140 mutant. Paralyzed individuals were recorded daily.

Age pigments, including lipofuscin and advanced glycation end-products, are endogenous fluorescent compounds that accumulate in the intestine with age across phyla [27]. High levels of age pigment indicate a physiologically aged state; thus, age pigments are valid reporters of nematode healthspan [27]. Similar results were observed in both methods used to quantify the autofluorescence of N2 worms during aging. The relative fluorescence of age pigments observed with fluorescence microscopy was significantly decreased after treatment with carnosol at various stages of life (Figures 5(a) and 5(b)). Similarly, according to the results obtained by the microplate reader, age pigment fluorescence was also significantly decreased in the carnosol-treated group compared with the control group (Figure 5(c)).

The current prevalence of obesity has become a global problem that impairs healthspan [28]. As shown in Figure 5(d), there was no significant difference in the intensity of Oil Red O staining between the control group and the carnosol treatment group. According to the quantification by ImageJ, we conclude that carnosol did not inhibit the fat accumulation of *C. elegans* (*p* > 0.05, ANOVA, Figure 5(e)).

The aggregation of misfolded proteins increases with age and leads to chronic proteotoxic stress, which is associated with various age-related neurodegenerative diseases, such as Alzheimer's disease and Huntington's disease [29]. We further investigated whether carnosol could also ameliorate internal proteotoxic stress in *C. elegans*. The following two *C. elegans* models of human neurodegenerative diseases were exploited in this study: CL4176 and AM140. The results showed that carnosol could significantly prolong the mean paralysis time induced by *β*-amyloid (A*β*) in the Alzheimer's disease model CL4176 by 21% in comparison with that of the control level (Figure 5(f) and Table 2). We also checked the potential of carnosol to slow the progression of Huntington's disease by using the AM140 *C. elegans* model. After treatment with carnosol, the polyQ-dependent paralysis of AM140 was also significantly delayed by 14% (Figure 5(g) and Table 2), indicating that carnosol could ameliorate detrimental effects during the development of age-related neurodegenerative diseases.

Taken together, these findings indicated that supplementation with carnosol could improve the healthspan of *C. elegans*, including improving mobility, reducing the formation of age pigments, and inhibiting the occurrence and development of neurodegenerative diseases, without affecting reproductive and fat deposition.

### 3.5. Genetic Requirements for the Enhanced Healthspan Induced by Carnosol Treatment in *C. elegans*

**Figure 6:**
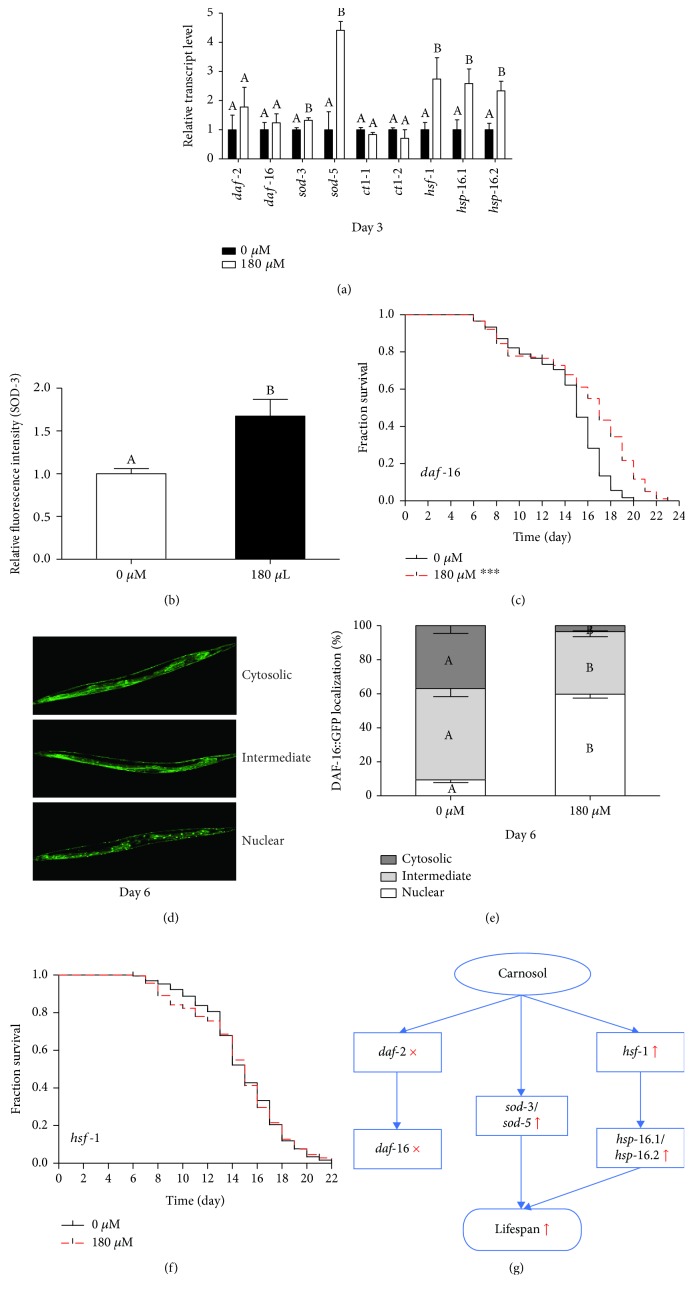
Mechanism of the carnosol-induced effects on antioxidant defense and longevity in *C. elegans*. (a) qRT-PCR was performed to investigate the expression of *hsp-16.1*, *hsp-16.2*, *sod-3*, *sod-5*, *ctl-1*, *ctl-2*, *daf-2*, *daf-16*, and *hsf-1*. (b) SOD-3::GFP expression in carnosol-treated worms was higher than that in control worms. (c) Carnosol treatment caused lifespan extension in the *daf-16*(mgDf50) mutant. (d) Representative images of the transgenic strain TJ356 with cytosolic, intermediate, and nuclear DAF-16::GFP localization. (e) Effect of carnosol on subcellular DAF-16 localization. (f) Carnosol did not extend lifespan in the *hsf-1*(sy441) I mutant. (g) Model of action of the carnosol-mediated lifespan extension in *C. elegans*.

Many genetic requirements and pathways that regulate lifespan have been identified in *C. elegans*. First, we considered the possibility that carnosol treatment acted as an effective antioxidant. The effects of carnosol on the expression of several antioxidant enzyme genes, including superoxide dismutases (SODs) (*sod-3* and *sod-5*) and catalases (*ctl-1* and *ctl-2*), were examined. As shown in Figure 6(a), the expression of *sod-3* and *sod-5* was significantly increased in the carnosol treatment group compared with the control group, while no significant effect of carnosol on *ctl-1* and *ctl-2* expression was observed. However, whether carnosol treatment might increase the expression levels of *ctl-1* and *ctl-2* in the middle or late stage will be investigated in the future. In addition, we quantified the relative expression of GFP in the SOD-3::GFP transgenic reporter strain CF1553. The mean GFP intensity of CF1553 was higher in the carnosol treatment group than in the control group (*p* < 0.05, ANOVA, Figure 6(b)). This result was consistent with the results of the upregulated expression of *sod-3*. Overall, the longevity-enhancing properties mediated by carnosol were associated with the increased expression of antioxidant enzyme genes.

Furthermore, it is well known that the insulin/insulin-like growth factor signaling (IIS) pathway is highly conserved and central to the growth and metabolism of diverse species; this pathway exerts its effect on lifespan by regulating a wide variety of cellular stress responses [30]. In *C. elegans*, the IIS signaling receptor DAF-2 ultimately directs the related kinases AKT-1, AKT-2, and SGK-1 to phosphorylate the FOXO protein DAF-16, thereby inhibiting its accumulation in the nucleus [31, 32]. DAF-16 is a key regulator of longevity, and its nuclear localization is a necessary prerequisite for the transcriptional activation of a broad spectrum of target genes, including antioxidant enzymes such as SOD-3 [33, 34]. By using transgenic strains that express the fused proteins DAF-16::GFP, we examined the translocation of DAF-16 to investigate the role of DAF-16 in modulating carnosol-induced longevity [33]. The localization of DAF-16 in untreated control worms was predominantly cytosolic and intermediate (Figure 6(e)). Treatment with carnosol for 6 days showed increased nuclear localization of DAF-16, with nuclear (60% ± 2%), intermediate (37% ± 3%), and cytosolic (3% ± 3%) localization phenotypes (Figure 6(e)). These results indicated that carnosol affected the subcellular distribution of DAF-16 and caused the translocation of DAF-16 from the cytoplasm to nuclei. To ascertain the effect of carnosol on the IIS pathway, we used qPCR to analyze the mRNA expression levels of key genes in the IIS pathway, including *daf-2* and *daf-16*. Strikingly, there was no significant change in the expression of *daf-2* and *daf-16* (Figure 6(a)). To further investigate whether carnosol increased lifespan by acting through the IIS pathway, we carried out a survival assay using mutant worms that lack the *daf-16* gene. As shown in Figure 6(c), compared with the control, carnosol also significantly extended the lifespan of *daf-16*(mgDf50) null mutations (*p* < 0.0001 as determined by the log-rank test). Considering that the expression levels of *daf-16* and *daf-2* were not altered by carnosol and that the loss of function of DAF-16 did not completely abolish the protective effect of carnosol, *daf-16* might be nonessential in the lifespan regulation mediated by carnosol. We speculated that carnosol might induce DAF-16 nuclear localization but did not increase its transcriptional activity. Therefore, the subcellular localization of DAF-16 activated by carnosol might not be the reason for the upregulation of *sod-3*. In fact, many antioxidant polyphenols had been found to regulate DAF-16 translocation, but the described longevity effects did not depend on DAF-16 activity [35, 36]. It was supported by other data indicating that DAF-16 translocation alone was not sufficient for its transcription-activating activity and that multiple interacting partners were necessary for proper DAF-16 activity in *C. elegans*, like 14-3-3 proteins and SIR-2.1 (the *C. elegans* ortholog of human Sirtuin 1) [37, 38]. Moreover, the subcellular distribution of DAF-16 was controlled by a variety of signaling pathways, including the DAF-7/transforming growth factor-*β* signaling and the c-Jun N-terminal kinase signaling [38]. Taken together, these data indicated that the carnosol-mediated extension of longevity was not associated with the IIS pathway.

In addition, we investigated the gene expression of heat shock transcription factor-1 (HSF-1), which is required for enhanced thermotolerance, proteotoxicity suppression, and lifespan extension [39]. A decrease in *hsf-1* activity accelerates tissue aging and shortens lifespan, while *hsf-1* overexpression prolongs lifespan [40]. The results showed that the mRNA level of *hsf-1* was significantly increased by 2.74 times in the carnosol treatment group compared with the control group (*p* < 0.05, ANOVA, Figure 6(a)). Furthermore, we also investigated the expression of heat shock protein genes (*hsp-16.1* and *hsp-16.2*) downstream of *hsf-1*. The expression of *hsp-16.1* and *hsp-16.2* was also more than doubled (Figure 6(a)). The increase in heat shock protein expression is beneficial not only to improve stress resistance under heat stress and other stresses but also to reduce the aggregation of proteins in age-related diseases during aging [40]. Therefore, the role of carnosol in delaying the development and progression of neurodegenerative diseases might be positively correlated with the upregulation of *hsp-16.1* and *hsp-16.2*. To further confirm that carnosol could activate the HSF-1 signaling pathway, we measured the lifespan of the *hsf-1* null mutant. It was found that the lifespan of *hsf-1* mutants was unchanged following carnosol treatment (*p* = 0.8294 as determined by the log-rank test, Figure 6(f)), suggesting that *hsf-1* was essential for carnosol-induced lifespan extension.

Taken together, the longevity benefit induced by carnosol in *C. elegans* might be related to increased expression of antioxidant enzyme genes and might be mediated by the HSF-1 pathway rather than dependent on the IIS pathway (Figure 6(g)). Therefore, the longevity mechanism regulated by carnosol might be mediated by upregulating the antioxidant enzyme genes (*sod-3* and *sod-5*) and activating heat shock protein expression (*hsp-16.1* and *hsp-16.2*) via the HSF-1 signaling pathway in *C. elegans* (Figure 6(g)).

## 4. Conclusions

In summary, carnosol was able to significantly decrease ROS accumulation in *C. elegans*. Carnosol also exhibited a strong stimulating effect on antioxidant enzyme activities and a marked inhibitory effect on MDA. Moreover, carnosol significantly increased the survival rate under normal and stress conditions. Moreover, lifespan extension was accompanied by health benefits, including improved mobility and neuroprotection and decreased age pigment accumulation, without inhibiting fertility and fat accumulation in *C. elegans*. Further research in pursuit of the molecular mechanism showed that carnosol enhanced antioxidant activity and stress tolerance and prolonged the healthspan by upregulating *sod-3*, *sod-5*, *hsf-1*, *hsp-16.1*, and *hsp-16.2*. However, no changes in the expression of the genes *daf-2*, *daf-16*, *clt-1*, and *clt-2* were induced. Carnosol significantly induced nuclear DAF-16::GFP localization in the TJ356 and also significantly extended lifespan of *daf-16* mutants. Finally, carnosol-mediated longevity was dependent on the *hsf-1* gene but not on the *daf-16* gene. Overall, the mechanism of action underlying carnosol-induced longevity was the improved healthspan mediated by the upregulation of antioxidant enzyme (*sod-3* and *sod-5*) and stress resistance gene *hsf-1* which further activated downstream *hsp-16.1* and *hsp-16.2* expression in *C. elegan*s (Figure 6(g)). Since carnosol is derived from natural plant polyphenols, we believe it has great potential to promote healthspan and prevent multiple age-related diseases in mammals.

## Figures and Tables

**Table 1:** Statistical analysis of the survival time of *C. elegans.*

Genotype	Treatment^1^	Mean time^2^	Median time^3^	Maximum time^4^	% effect^5^	*p* value^6^	Uncensored/*n*
N2	0 *μ*M	20.13 ± 0.23^a^	20.33 ± 0.58^a^	29.67 ± 1.53^a^	—	—	159/180
	180 *μ*M	23.96 ± 0.89^b^	22.67 ± 1.15^b^	37.33 ± 5.03^b^	19	<0.0001	159/180
	0 *μ*M (paraquat)	5.29 ± 0.15^a^	5.17 ± 0.29^a^	8.50 ± 0.00^a^	—	—	134/180
	180 *μ*M (paraquat)	6.38 ± 0.03^b^	6.00 ± 0.00^b^	10.50 ± 0.00^b^	21	<0.0001	142/180
	0 *μ*M (35°C)	16.95 ± 0.14^a^	18.00 ± 0.00^a^	22.33 ± 0.58^a^	—	—	180/180
	180 *μ*M (35°C)	18.45 ± 0.15^b^	19.00 ± 0.00^b^	23.00 ± 0.00^b^	9	<0.0001	180/180

*daf-16*	0 *μ*M	13.95 ± 0.10^a^	15.00 ± 0.00^a^	19.33 ± 0.58^a^	—	—	137/180
	180 *μ*M	15.57 ± 0.14^b^	17.00 ± 0.00^b^	22.33 ± 0.58^b^	12	<0.0001	142/180

*hsf-1*	0 *μ*M	14.33 ± 0.29^a^	13.00 ± 0.00^a^	21.00 ± 1.15^a^	—	—	151/180
	180 *μ*M	14.01 ± 0.20^a^	13.38 ± 0.48^a^	21.75 ± 0.50^a^	—	0.8294	143/180

^1^The control groups for stress resistance and the lifespan of mutants (*daf-16* and *hsf-1*) were referred to previously published data [12]. ^2^Mean survival time = (1/*n*)∑_*j*_((*x*_*j*_ + *x*_*j*+1_)/2)*dj*, where *j* is the age category, *d*_*j*_ is the number of worms that died in the age interval (*x*_*j*_, *x*_*j*_ + 1), and *n* is the total number of worms. ^3^The median lifespan is the time at which the percentage of surviving worms equals 50%. ^4^The maximum lifespan is the time at which survival equals 0%. ^5^% effect was calculated by (*T*‐*C*)/*C* ^∗^100, where *T* is the mean survival time of worms treated with carnosol and *C* is the mean survival time of the control. ^6^The *p* value was calculated using the log-rank test by comparing the carnosol-treated group with the control.

**Table 2:** Statistical analysis of the paralysis time of *C. elegans*.

Genotype	Treatment^1^	Mean time^2^	Median time^3^	Maximum time^4^	% effect^5^	*p* value^6^	Uncensored/*n*
CL4176	0 *μ*M (25°C)	10.94 ± 0.28^a^	12.00 ± 0.00^a^	16.00 ± 0.00^a^	—	—	180/180
	180 *μ*M (25°C)	13.29 ± 0.21^b^	14.00 ± 0.00^b^	18.00 ± 0.00^b^	21	<0.0001	180/180

AM140	0 *μ*M	12.27 ± 0.09^a^	12.00 ± 0.00^a^	17.00 ± 0.00^a^	—	—	155/180
	180 *μ*M	13.94 ± 0.18^b^	13.17 ± 0.29^b^	20.67 ± 0.58^b^	14	<0.0001	139/180

^1^The control groups for paralysis assays were referred to previously published data [12]. ^2^Mean paralysis time = (1/*n*)∑_*j*_((*x*_*j*_ + *x*_*j*+1_)/2)*dj*, where *j* is the age category, *d*_*j*_ is the number of worms that paralyzed in the age interval (*x*_*j*_, *x*_*j*_ + 1), and *n* is the total number of worms. ^3^The median paralysis is the time at which the percentage of nonparalytic worms equals 50%. ^4^The maximum paralysis is the time at which paralysis equals 0%. ^5^% effect was calculated by (*T*‐*C*)/*C* ^∗^100, where *T* is the mean paralysis time of worms treated with carnosol and *C* is the mean paralysis time of control. ^6^The *p* value was calculated using the log-rank test by comparing the carnosol-treated group with the control.

## Data Availability

The data used to support the findings of this study are included within the article and the supplementary information file.

## Abbreviations

A*β*: Alzheimer *β*-amyloid
CAT: Catalase
*C. elegans*: *Caenorhabditis elegans*
*E. coli* OP50: *Escherichia coli strain* OP50
GSH-Px: Glutathione peroxidase
H_2_DCF-DA: 2,7-Dichlorodihydrofluorescein diacetate
HSF-1: Heat shock transcription factor-1
IIS: Insulin/insulin-like growth factor signaling
MDA: Malondialdehyde
mL: Milliliter
mol: Molar
NGM: Nematode growth medium
PolyQ: Polyglutamine
qRT-PCR: Quantitative reverse transcription-polymerase chain reaction
ROS: Reactive oxygen species
SOD: Superoxide dismutase
*μ*L: Microliter.

## Supplementary Materials

Supplementary Materials Table S1: primer sequences for qRT-PCR analysis.
