# Supplementary material for: Carnosol Improved Lifespan and Healthspan by Promoting Antioxidant Capacity in Caenorhabditis elegans
Source: Oxid Med Cell Longev. 2019 Jun 24;2019:5958043. doi: 10.1155/2019/5958043 (PMC6612998; doi:10.1155/2019/5958043)
Supplement: Supplementary Materials — Table S1: primer sequences for qRT-PCR analysis. [file 5958043.f1.docx]

**Supplementary Information**

**Carnosol improved lifespan and healthspan by promoting antioxidant capacity in *Caenorhabditis elegans***

Chunxiu Lin^a^**^†^**, Xiaoying Zhang^a^**^†^**, Zuanxian Su^b^, Jie Xiao^a^, Muwen Lv^a^, Yong Cao^a^ and Yunjiao Chen^a^*

**Table S1 Primer sequences for** **qRT-PCR analysis**

| **Gene** | **Primer** |
| --- | --- |
| *hsp-16.1* | CTGAATCTTCTGAGATTGTTAAC (F)  TTTGTTCAACGGGCGCTTGC (R) |
| *hsp-16.2* | CTGCAGAATCTCTCCATCTGAGTC (F)  AGATTCGAAGCAACTGCACC (R) |
| *sod-3* | CCAACCAGCGCTGAAATTCAATGG (F)  GGAACCGAAGTCGCGCTTAATAGT (R) |
| *sod-5* | GAACTGCTGTCTTCGGAACTG (F)  CCATGAAGTCCTGGTGACAAT (R) |
| *ctl-1* | GCGGATACCGTACTCGTGAT (F)  GTGGCTGCTCGTAGTTGTGA (R) |
| *ctl-2* | GAGAATGTGCCAGAACTTTGC (F)  CTTGACACGAGCTCCAAAATC (R) |
| *daf-2* | GGATAAAGGCGAATCAAAGTGTC (F)  CGATACACTTTCCCTTGTGATAGAC (R) |
| *daf-16* | CTTCAAGCCAATGCCACTACC (F)  GGAGATGAGTTGGATGTTGATAGC (R) |
| *hsf-1* | TTGACGACGACAAGCTTCCAGT (F)  AAAGCTTGCACCAGAATCATCCC (R) |
| *act-1* | TCCAAGAGAGGTATCCTTAC (F)  CGGTTAGCCTTTGGATTGAG (R) |
